# Intrinsic Susceptibility MRI Identifies Tumors with *ALK^F1174L^* Mutation in Genetically-Engineered Murine Models of High-Risk Neuroblastoma

**DOI:** 10.1371/journal.pone.0092886

**Published:** 2014-03-25

**Authors:** Yann Jamin, Laura Glass, Albert Hallsworth, Rani George, Dow-Mu Koh, Andrew D. J. Pearson, Louis Chesler, Simon P. Robinson

**Affiliations:** 1 Division of Radiotherapy and Imaging, The Institute of Cancer Research and Royal Marsden NHS Foundation Trust, London, United Kingdom; 2 Division of Cancer Therapeutics, The Institute of Cancer Research, London, United Kingdom; 3 Division of Molecular Pathology, The Institute of Cancer Research, London, United Kingdom; 4 Division of Clinical Studies, The Institute of Cancer Research, London, United Kingdom; 5 Department of Pediatric Haematology and Oncology, Dana-Farber Cancer Institute and Children's Hospital Boston, Harvard Medical School, Boston, Massachusetts, United States of America; University of Michigan School of Medicine, United States of America

## Abstract

The early identification of children presenting *ALK^F1174L^*-mutated neuroblastoma, which are associated with resistance to the promising *ALK* inhibitor crizotinib and a marked poorer prognosis, has become a clinical priority. In comparing the radiology of the novel Th-*ALK^F1174L^*/Th-*MYCN* and the well-established Th-*MYCN* genetically-engineered murine models of neuroblastoma using MRI, we have identified a marked *ALK^F1174L^*-driven vascular phenotype. We demonstrate that quantitation of the transverse relaxation rate R_2_* (s^−1^) using intrinsic susceptibility-MRI under baseline conditions and during hyperoxia, can robustly discriminate this differential vascular phenotype, and identify *MYCN*-driven tumors harboring the *ALK^F1174L^* mutation with high specificity and selectivity. Intrinsic susceptibility-MRI could thus potentially provide a non-invasive and clinically-exploitable method to help identifying children with *MYCN*-driven neuroblastoma harboring the *ALK^F1174L^* mutation at the time of diagnosis.

## Introduction

Neuroblastoma arises in the sympathetic nervous system during embryogenesis and is the most common extracranial solid tumor in children [1]. For the established subset of patients presenting with high-risk neuroblastoma, the current portfolio of therapeutic options has limited success, with five-year survival rates rarely exceeding 40%.

The poor clinical outcome and aggressive tumor phenotype of high-risk neuroblastoma strongly correlates with amplification of the proto-oncogene *MYCN* and enhanced tumor angiogenesis [2]. Recently, mutations in the anaplastic lymphoma kinase (*ALK*) tyrosine kinase gene have been identified in ∼8–10% of primary neuroblastoma, leading to constitutive activation of the ALK protein. The most common and potent *ALK* mutation, *ALK^F1174L^*, is associated preferentially with *MYCN* amplification, a markedly poorer prognosis, and confers resistance to the promising *ALK* inhibitor crizotinib [3]–.

With crizotinib in pediatric phase I clinical trials, and other *ALK* inhibitor studies in development, a current challenge is to rapidly identify upfront children with high-risk *ALK* mutated or amplified neuroblastoma who may benefit from or become resistant to *ALK*-targeted therapy.

As most pediatric cancers, including neuroblastoma, originate from only a few genetic anomalies during development, they are amenable to genetically engineered mouse (GEM) modeling approaches. GEM models of neuroblastoma, such as the Th-*MYCN* murine model, which develop spontaneous tumors mirroring the major pathophysiological, genetic and radiological features of high-risk *MYCN*-amplified childhood neuroblastoma, represent clinically-relevant tools for the study of neuroblastoma biology and response to novel therapeutics [6]–[8]. The development of GEM models co-expressing *ALK^F1174L^* and *MYCN* to the neural crest, such as the Th-*ALK^F1174L^*/Th-*MYCN* model, have recently been used to demonstrate how the *ALK^F1174L^* mutation potentiates the oncogenic activity of *MYCN*, and that *ALK^F1174L^* acquired resistance to crizotinib can be overcome through inhibition of key cellular modulators of *n-myc* [9]–[11].

In this study, we hypothesized that the presence of the *ALK^F1174L^* mutation results in a phenotypic difference in hemodynamic vasculature in tumors in the Th-*ALK^F1174L^*/Th-*MYCN* model, and which can be evaluated using intrinsic susceptibility magnetic resonance imaging (MRI). The aim of our study was to demonstrate that quantitation of the transverse relaxation rate, R_2_*, and changes in R_2_* induced by breathing 100% oxygen, ΔR_2_*_oxygen-air_, could discriminate between tumors arising in Th-*ALK^F1174L^*/Th-*MYCN* and Th-*MYCN* mice, and that intrinsic susceptibility MRI could thus potentially provide a non-invasive and clinically-translatable method to help identify children, presenting *MYCN*-amplified neuroblastoma harboring the *ALK^F1174L^* mutation.

## Materials and Methods

### Ethics statement

All procedures involving animals were approved by the Institute of Cancer Research Animal Ethics Committee and the UK Home Office and carried out according to the United Kingdom National Cancer Research Institute guidelines for the welfare of animals in cancer research [12].

### Animal models

The generation of the Th-*ALK^F1174L^*/Th-*MYCN* mice has been recently described [11]. Th-*ALK^F1174L^*/Th-*MYCN* and Th-*MYCN* mice were identified by analyzing DNA from mice tails using real-time quantitative reverse transcription polymerase chain reaction (qRT-PCR, Transnetyx Inc., Cordova, Tennessee), and mice with tumors were initially identified by palpation.

### Magnetic Resonance Imaging

All the ^1^H MRI studies were performed on a 7T Bruker horizontal bore micro-imaging system (Bruker Instruments, Ettlingen, Germany) using a 3 cm birdcage coil. Anesthesia was induced by an intraperitoneal 0.1 ml injection of a combination of fentanyl citrate (0.315 mg/ml) plus fluanisone (10 mg/ml) (Hypnorm, Janssen Pharmaceutical, Oxford, UK) and midazolam (5 mg/ml) (Roche, Welwyn Garden City, UK) and water (1∶1∶2). A nose piece was positioned for oxygen delivery.

For all the mice, anatomical T_2_-weighted coronal and transverse images were acquired from twenty contiguous 1 mm-thick slices through the mouse abdomen, using a rapid acquisition with refocused echoes (RARE) sequence with 4 averages of 128 phase encoding steps over a 3×3 cm field of view, two echo times (TE) of 36 and 132 ms, a repetition time (TR) of 4.5 s and a RARE factor of 8. These images were used to determine tumor volumes, and for planning the intrinsic-susceptibility MRI measurements, which included optimization of the local field homogeneity. The baseline transverse relaxation rate R_2_*, sensitive to the concentration of paramagnetic species, principally deoxyhemoglobin, was quantified in tumors from Th-*ALK^F1174L^*/Th-*MYCN* (n = 23) and Th-*MYCN* (n = 21) mice, using a multiple gradient echo (MGE) sequence. MGE images were acquired from three 1 mm thick transverse slices through each tumor, using 8 averages of 128 phase encoding steps over a 3×3 cm field of view, and an acquisition time of 3 min 20 s. Images were acquired using 8 echoes spaced 3 ms apart, an initial echo time of 6 ms, a flip angle α = 45° and a repetition time of 200 ms. Subsequently, 100% oxygen (BOC Ltd, Guildford, UK) was delivered at a rate of 2 L/min to Th-*ALK^F1174L^*/Th-*MYCN* (n = 10) and Th-*MYCN* (n = 12) mice. After a 5 minutes equilibrium period, identical MGE images were acquired whilst the mouse continued to inhale oxygen.

All the MGE data were fitted voxelwise using in-house software (ImageView, working under IDL, ITT, Boulder, Colorado, USA) with a robust Bayesian approach that provided estimates of R_2_* and ΔR_2_*_oxygen-air_ ( = R_2_*_oxygen_−R_2_*_air_) [13].

### Histological Assessment

Formalin fixed paraffin embedded sections from Th-*ALK^F1174L^*/Th-*MYCN* and Th-*MYCN* mice were stained with haematoxylin and eosin, and visualized under light microscopy. The extent of functional vasculature in tumors from both models was assessed using the perfusion marker Hoechst 33342, and quantified as fluorescent area fractions (%), as previously described [8].

### Statistical analysis

Statistical analysis was performed using GraphPad Prism 5 (GraphPad Software Inc., La Jolla, USA). The mean values for tumor volume, mean of median values for R_2_* and ΔR_2_*_oxygen-air_, and the mean fluorescent area fractions were used for statistical analysis. R_2_* and ΔR_2_*_oxygen-air_ were assumed to be normally distributed, which was confirmed using the D'Agostino-Pearson omnibus K^2^ normality test [14]. Any significant difference in tumor volume, R_2_*, ΔR_2_*_oxygen-air_ and the fluorescent area fractions between Th-*ALK^F1174L^*/Th-*MYCN* and Th-*MYCN* mice were identified using Student's 2-tailed unpaired t-test, with a 5% level of significance.

## Results

### ALK^F1174L^mutation induces a differential radiological presentation of neuroblastoma

**Figure 1:**
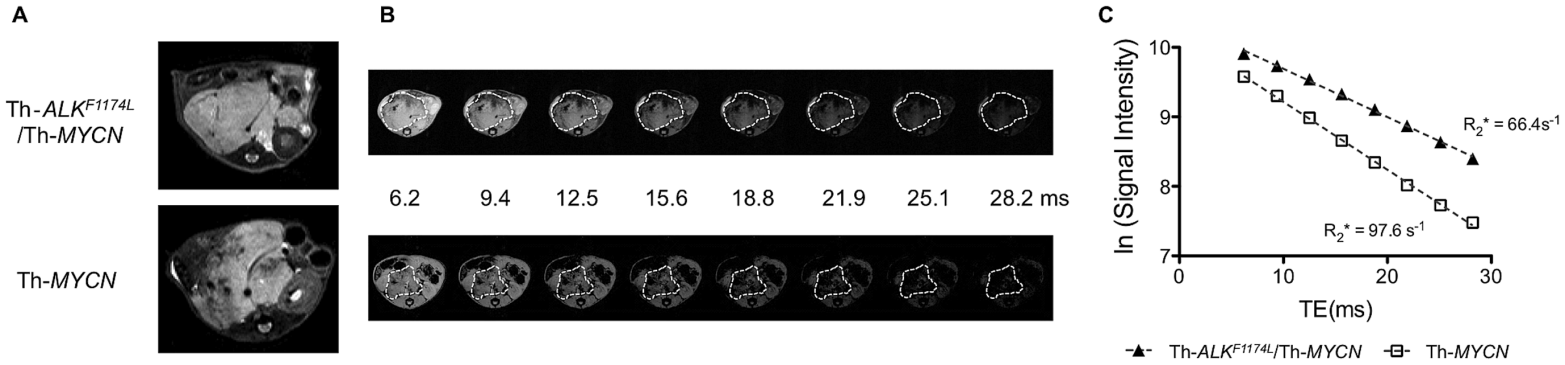
Radiological comparison of the Th-*ALK^F1174L^*/Th-*MYCN* and Th-*MYCN* mice with abdominal neuroblastoma. **A**) Anatomical transverse T_2_-weighted MR images acquired with a rapid acquisition with refocused echoes (RARE) sequence and **B**) anatomical transverse T_2_*-weighted MR images acquired at increasing gradient echo times as indicated, from representative presenting with abdominal neuroblastoma. **C**) Note the rapidly decaying tumor signal intensity in the Th-MYCN mouse, compared to the more sustained tumor signal observed in the Th-*ALK^F1174L^*/Th-*MYCN* mouse.

**Figure 2:**
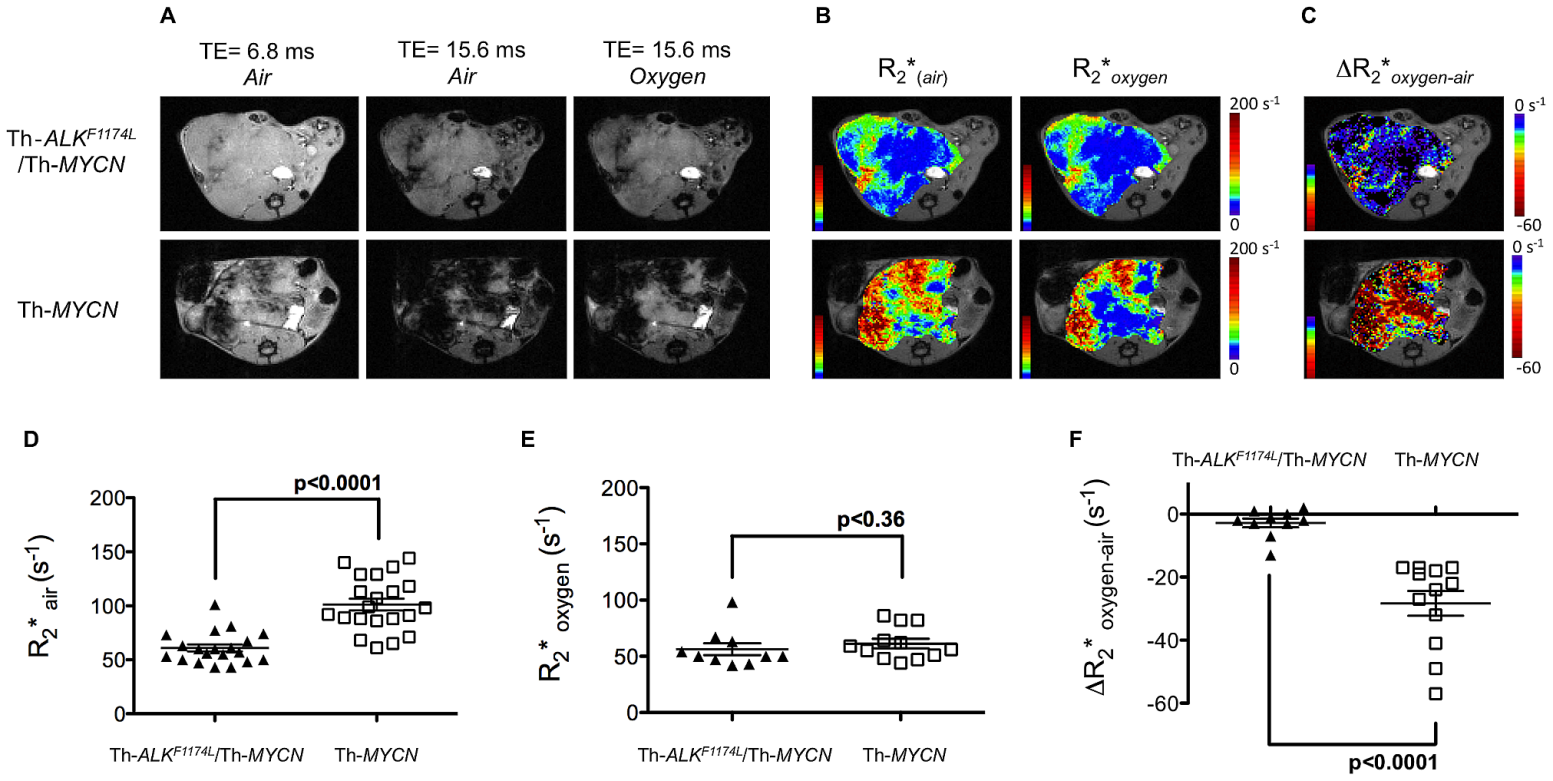
Identification of tumors harboring the *ALK^F1174L^* mutation in *MYCN*-driven transgenic mice with intrinsic susceptibility MRI. **A**) Anatomical transverse T_2_*-weighted MR images acquired at TE = 6.8 and 15.6 ms from representative Th-*ALK^F1174L^*/Th-*MYCN* and Th-*MYCN* mice with abdominal neuroblastoma during initial air breathing, and at TE = 15.6 ms after 5 minutes of continuous inhalation of 100% oxygen. **B**) Corresponding parametric tumor transverse relaxation rate R_2_* maps calculated during initial air breathing and after 3 minutes of continuous inhalation of 100% oxygen. **C**) Resulting parametric tumor ΔR_2_*_oxygen-air_ (R_2_*_oxygen_−R_2_*_air_) maps. **D**) Tumor R_2_* during initial air breathing, **E**) tumor R_2_* after 5 minutes of breathing 100% oxygen, and **F**) tumor ΔR_2_*_oxygen-air_ (R_2_*_oxygen_−R_2_*_air_) were determined from Th-*ALK^F1174L^*/Th-*MYCN* and Th-*MYCN* mice with abdominal neuroblastoma. Individual data points represent the mean of the median values determined from all three imaging slices for each animal, as well as the mean ±1 s.e.m, p, Student's 2-tailed unpaired t-test with a 5% level of significance.

Anatomical T_2_-weighted RARE MR images revealed solid masses within the retroperitoneum in peri-renal and para-spinal abdominal regions of both the Th-*ALK^F1174L^*/Th-*MYCN* and Th-*MYCN* mice, typical of the clinical distribution and radiological presentation of human neuroblastoma (Figure 1A) [15], [16]. All the Th-*ALK^F1174L^*/Th-*MYCN* (n = 23) and Th-*MYCN* (n = 21) mice examined presented with abdominal tumors with wide range of volumes (525–3400 mm^3^ for tumors in Th-*ALK^F1174L^*/Th-*MYCN* mice and 335–2450 mm^3^ for tumors in Th-*MYCN* mice). Tumors in the Th-*MYCN* mice appeared heterogeneous, with 94% of tumors presenting with areas of hypointense signal, consistent with previous observations [8]. Tumors in the Th-*ALK^F1174L^*/Th-*MYCN* mice were generally hyperintense and significantly more homogeneous, with only 48% of the tumors having hypointense regions (p = 0.006, χ^2^-test). On T_2_*-weighted images (Figure 1B and C, and Figure 2A), tumors in the Th-*MYCN* mice were generally hypointense at longer echo times, whereas the signal from tumors in the Th-*ALK^F1174L^*/Th-*MYCN* remained hyperintense, providing a stark positive contrast with the surrounding organs for 74% of the Th-*ALK^F1174L^*/Th-*MYCN* mice (compared to 14% for the Th-*MYCN* mice, p<0.001, χ^2^-test).

### ALK^F1174L^mutation results in a slower tumor transverse relaxation rate, R_2_^*^, in neuroblastoma

Parametric maps revealed a heterogeneous distribution of R_2_* in tumors in both models (Figure 2B). Quantitation of the transverse relaxation R_2_* revealed significantly slower rates in tumors in the Th-*ALK^F1174L^*/Th-*MYCN* cohort, compared to the faster R_2_* rate determined in tumors in the Th-*MYCN* mice (Figure 2D). Baseline R_2_* detected tumors harboring the *ALK^F1174L^* mutation with a sensitivity of 90% (95% CI: 66.9–98.2) and a specificity of 81% (95% CI: 57.4–93.7).

### ALK^F1174L^mutation is associated with a stark differential BOLD MRI response to hyperoxic challenge in neuroblastoma

Continuous inhalation of 100% oxygen resulted in tumors in the Th-*MYCN* mice demonstrating a very strong, heterogeneous increase in T_2_*-weighted image signal intensity (blood oxygen level dependent (BOLD) effect) (Figure 2A). In contrast, tumors from Th-*ALK^F1174L^*/Th-*MYCN* mice showed a negligible BOLD effect on T_2_* weighted image intensity with hyperoxia. As with baseline R_2_*, parametric maps revealed a heterogeneous distribution of ΔR_2_*_oxygen-air_ in tumors in both models (Figure 2B and C). Tumor regions showing a relatively fast baseline R_2_* remained so during hyperoxia, whereas regions of relatively slower baseline R_2_* typically showed a more marked reduction in R_2_* with 100% O_2_. The significant difference in R_2_* between the two models was lost with 100% oxygen challenge (Figure 2E). As a consequence, tumors in Th-*ALK^F1174L^*/Th-*MYCN* mice demonstrated a significantly lower absolute value of ΔR_2_*_oxygen-air_ than the tumors in Th-*MYCN* mice (Figure 2F). Hyperoxia-induced ΔR_2_*_oxygen-air_ detected tumors harboring the *ALK^F1174L^* mutation with a sensitivity of 90% (95% CI: 54.1–99.5) and a specificity of 94.1% (95% CI: 69.2–99.7).

No correlation between tumor R_2_* and ΔR_2_*_oxygen-air_, or between either R_2_* or ΔR_2_*_oxygen-air_ and tumor volume, was determined across the cohorts, indicating that the differences in intrinsic susceptibility MRI between tumors in the Th-*ALK^F1174L^*/Th-*MYCN* and Th-*MYCN* mice were independent of tumor size.

### ALK^F1174L^mutation is associated with reduced functional vasculature in neuroblastoma

**Figure 3:**
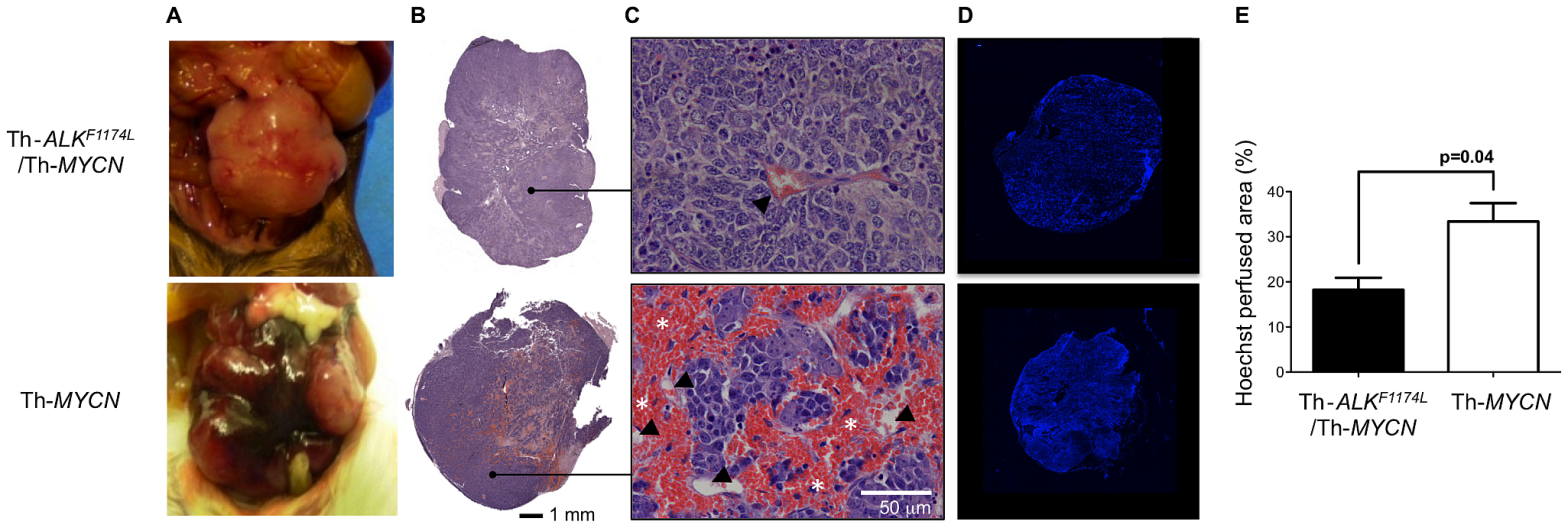
Pathological comparison of tumors from Th-*ALK^F1174L^*/Th-*MYCN* and Th-*MYCN* mice with abdominal neuroblastoma. **A**) Gross pathology, **B**) composite images, and **C**) high magnification (x200) images from hematoxylin and eosin stained sections. Note the presence of large hemorrhagic regions filled with aggregated erythrocytes (*, blood lakes) extravasated from blood vessels (arrowed) in the tumor from the Th-*MYCN* mouse. **D**) Composite fluorescence images of uptake of the perfusion marker Hoechst 33342 into tumors from Th-*ALK^F1174L^*/Th-*MYCN* and Th-*MYCN* mice with abdominal neuroblastoma. **E**) Quantitation of Hoechst 33342 uptake revealed significantly lower functionally perfused vasculature in tumors of Th-*ALK^F1174L^*/Th-*MYCN* mice (n = 5) compared with tumors in Th-*MYCN* mice (n = 5). Data are mean ±1 s.e.m, p, Student's 2-tailed unpaired t-test with a 5% level of significance.

Gross examination of the tumors *in situ*, prior to excision, revealed an intense dark red coloration across the whole tumor in the Th-*MYCN* mice, in contrast to the pale appearance of tumors in the Th-*ALK^F1174L^*/Th-*MYCN* GEM model (Figure 3A). Histological examination with H&E staining revealed the presence of large hemorrhagic regions filled with stacked erythrocytes in 100% of the tumors from Th-*MYCN* mice (n = 10), but only in 10% the Th-*ALK^F1174L^*/Th-*MYCN* model (n = 10, p<0.0001, χ^2^-test) (Figure 3B and C). Fluorescence microscopy of Hoechst 33342 uptake revealed homogeneously and well-vascularized tumors in both models (Figure 3D). However, quantitation of the fluorescent area fractions revealed that tumors in the Th-*MYCN* mice had significantly higher uptake, consistent with the presence of more perfused, functional vasculature (Figure 3E).

## Discussion

In the current study we demonstrate that baseline R_2_* and hyperoxia-induced ΔR_2_* can discriminate a differential hemodynamic tumor vascular phenotype between tumors arising in Th-*ALK^F1174L^*/Th-*MYCN* and Th-*MYCN* models of high-risk, *MYCN* over-expressing neuroblastoma. With a sensitivity of 90% and a specificity of 81% for baseline R_2_*, and a sensitivity of 90% and a specificity of 94% for 100% oxygen-induced ΔR_2_*, intrinsic susceptibility MRI provides a robust method to discriminate and identify Th-*MYCN* transgenic mice harboring the *ALK^F1174L^* mutation.

The rapid and completely noninvasive quantitation of tumor R_2_* is suitable for the scanning of young children. Furthermore, such pediatric imaging sessions are usually performed under general anesthesia, providing an opportunity to transiently perturb the oxygen content inhaled by the patient, and enabling quantitation of tumor R_2_* under more hyperoxic conditions (from 21 to 30–40% O_2_). Upon successful translation, intrinsic susceptibility MRI could provide a rapid method to identify children with *ALK*-driven tumors, enabling the stratification of children with this ultra high-risk neuroblastoma at the time of diagnosis.

The baseline transverse relaxation rate R_2_* is sensitive to the concentration of paramagnetic deoxyhemoglobin in the vascular compartment in tissue (BOLD effect). Compared to most normal tissues, tumors exhibit relatively fast native R_2_* values, a consequence of the high concentration of deoxygenated erythrocytes within the vascular compartment associated with immature, irregular and unstable microcirculation [17]. The fast baseline tumor R_2_* measured in the Th-*MYCN* model is consistent with the aggregation of deoxygenated erythrocytes, described as blood lakes [18], and which are characteristic of childhood neuroblastoma [19]. The significantly slower baseline R_2_* and differential intrinsic susceptibility MRI presentation of tumors in the Th-*ALK^F1174L^*/Th-*MYCN* mice is consistent with the absence of such blood lakes.

Inhalation of high oxygen content gases results in the rapid re-oxygenation of hemoglobin, with paramagnetic deoxyhemoglobin within perfused tumor vessels being replaced by diamagnetic oxyhemoglobin, and a reduction in R_2_* [20]. Hyperoxia resulted in an overall significant reduction in R_2_* of tumors in the Th-*MYCN* mice, whereas the response in tumors in the Th-*ALK^F1174L^*/Th-*MYCN* mice was negligible, suggesting a clear difference in hemodynamic functional vasculature between the two GEM models. This was corroborated by the significant overall difference in Hoechst 33342 uptake. Interestingly, a spatially different hyperoxia ΔR_2_* response was apparent in tumors in the Th-*MYCN* mice. Tumor regions exhibiting relatively fast baseline R_2_* showed a less pronounced response with 100% O_2_ breathing, also consistent with the presence of blood lakes which are typically disconnected from the perfused vascular network [21]. In contrast, tumor regions with relatively slower baseline R_2_* showed a marked response to hyperoxia, indicative of functional vasculature.

Intriguingly, tumors from the Th-*ALK^F1174L^*/Th-*MYCN* mice showed no clear ΔR_2_* response to hyperoxia, despite Hoechst 33342 uptake indicating the presence of perfused blood vessels in both GEM models. This suggests a difference in vascular architecture that precludes erythrocyte delivery, but not plasma perfusion, in the tumor vessels in the Th-*ALK^F1174L^*/Th-*MYCN* mice, or in oxygen consumption by the tumor cells [17], [22]. The extensive hemorrhage and blood lakes present in the Th-*MYCN* model are indicative of vessel wall instability due to rapid endothelial cell proliferation and defective pericyte coverage [23]. This implies that the tumor vasculature in the Th-*ALK^F1174L^*/Th-*MYCN* mice may be more mature.

Given its relationship to blood oxygen saturation and partial pressure of oxygen in and around blood vessels, quantitation of baseline tumor R_2_* is also being investigated as an imaging biomarker of hypoxia [24]. For example, slow baseline R_2_* has been shown to correlate with increased hypoxia, determined by pimonidazole staining, in chemically-induced rat mammary tumors [25]. Conversely, fast baseline R_2_* is associated with hypoxia in prostate cancer [26]. In this context, we have recently shown that neuroblastoma from Th-*MYCN* mice exhibiting fast baseline R_2_*, induced by a hemorrhagic phenotype, are relatively oxic, as revealed by negligible pimonidazole staining [8]. R_2_* is first of all a marker of impaired hemodynamic function and as such shares a causal relationship to hypoxia. However this relationship is ambivalent as several differential hemodynamic phenotypes can in fact lead to hypoxia, while having an opposite effect on R_2_* values. In this study the suggested tumor vascular phenotype in the Th-*ALK^F1174L^*/Th-*MYCN* model, which hinders the delivery of erythrocytes and causes a significantly slower R2*, may result in increased tumour hypoxia in the Th-*ALK^F1174L^*/Th-*MYCN* model compared with the Th-*MYCN* model.

Recent reports have implicated a role of the *ALK^F1174L^* mutation in tumor angiogenesis in neuroblastoma. Selective targeted inhibition of *ALK* resulted in a significant reduction in vascular density in xenografts derived from *MYCN* non-amplified SH-SY5Y cells, which harbor the *ALK^F1174L^* mutation, accompanied with a decrease in vascular endothelial growth factor (VEGF) and matrix metalloproteinases (MMPs) [27]. The expression of MMP-9 by stromal cells has previously been shown to regulate the vascular architecture in a murine orthotopic *MYCN*-amplified neuroblastoma xenograft model by promoting pericyte recruitment [28]. Collectively, these studies suggest that *ALK* and the *ALK^F1174L^* mutation contribute to tumor vasculogenesis and pericyte recruitment via the regulation of VEGF and MMP-9, leading to a dense vascular network with smaller, more stable vessels. The differential intrinsic susceptibility MRI vascular phenotype observed in tumors in the Th-*MYCN and* Th-*ALK^F1174L^*/Th-*MYCN* models reported herein demonstrates an important role for *ALK^F1174L^* in angiogenesis in *MYCN*-overexpressing neuroblastoma *in vivo*, strongly suggesting a role in vasculogenesis.

This study has some limitations. The Th-*ALK^F1174L^* mutation leads to the constitutive activation of the *ALK* protein in neuroblastoma [29]–[32], and the vascular phenotype associated with amplified or wild-type (WT) *ALK* expression is currently unknown. Amplification of *ALK* has also been shown to lead to activation of the *ALK* protein with, however, a 17-fold reduced kinase activity compared to *ALK^F1174L^* mutants [29]. In the absence of a Th-*ALK^wt^* GEM model, it is difficult to conclude if intrinsic susceptibility MRI would be solely able to identify *ALK^F1174L^*-mutated MYCN amplified neuroblastoma, or more generally *ALK*-driven MYCN amplified neuroblastoma.

There is a clear need to more deeply interrogate the role of the *ALK^F1174L^* mutation on vascular morphogenesis and architecture, which may be a major determinant of impaired drug delivery and a contributing factor to the poor prognosis of children with *ALK^F1174L^*-mutated *MYCN*-amplified neuroblastoma. The altered vascular phenotype may also impact on the response to anti-vascular therapies, including cediranib, currently being considered in clinical trials for the treatment of high-risk *MYCN*-amplified neuroblastoma [33]. Retrospective analysis of historical pathological samples for *ALK* mutations should provide sufficient statistical power to shed a light on the vascular phenotype of neuroblastoma associated with ALK amplification, and each of the rare ALK mutations, including the lethal *ALK^F1174L^* mutation.

Coupled with our recent identification of quantitation of R_2_* as a biomarker of treatment response to cediranib in the Th-*MYCN* GEM model [8], the present study reinforces R_2_* as a biomarker of vasculogenesis and its response to therapy in these clinically relevant GEM models. Furthermore, it provides a strong rationale for the evaluation of intrinsic susceptibility MRI for assessing any anti-angiogenic effects resulting from successful targeted inhibition of ALK signalling in *ALK^F1174L^* mutated neuroblastoma, which could ultimately used for the assessment of second generation ALK inhibitors [34], [35]. To conclude, this study has provided a strong rationale for the immediate incorporation of intrinsic susceptibility MRI into forthcoming imaging-embedded clinical trials of next generation *ALK* inhibitors in *ALK*-mutated and -amplified neuroblastoma.

## References

[1] Maris JM (2010) Recent advances in neuroblastoma. N Engl J Med 362: 2202–2211. 20558371 10.1056/NEJMra0804577 PMC3306838

[2] Meitar D, Crawford SE, Rademaker AW, Cohn SL (1996) Tumor angiogenesis correlates with metastatic disease, N-myc amplification, and poor outcome in human neuroblastoma. J Clin Oncol 14: 405–414. 8636750 10.1200/JCO.1996.14.2.405

[3] De Brouwer S, De Preter K, Kumps C, Zabrocki P, Porcu M, et al (2010) Meta-analysis of neuroblastomas reveals a skewed ALK mutation spectrum in tumors with MYCN amplification. Clin Cancer Res 16: 4353–4362. 20719933 10.1158/1078-0432.CCR-09-2660

[4] Sasaki T, Rodig SJ, Chirieac LR, Janne PA (2010) The biology and treatment of EML4-ALK non-small cell lung cancer. Eur J Cancer 46: 1773–1780. 20418096 10.1016/j.ejca.2010.04.002 PMC2888755

[5] Bresler SC, Wood AC, Haglund EA, Courtright J, Belcastro LT, et al (2011) Differential inhibitor sensitivity of anaplastic lymphoma kinase variants found in neuroblastoma. Science translational medicine 3: 108ra114. 10.1126/scitranslmed.3002950 PMC3319004 22072639

[6] Chesler L, Weiss WA (2011) Genetically engineered murine models—contribution to our understanding of the genetics, molecular pathology and therapeutic targeting of neuroblastoma. Semin Cancer Biol 21: 245–255. 21958944 10.1016/j.semcancer.2011.09.011 PMC3504935

[7] Weiss WA, Aldape K, Mohapatra G, Feuerstein BG, Bishop JM (1997) Targeted expression of MYCN causes neuroblastoma in transgenic mice. Embo J 16: 2985–2995. 9214616 10.1093/emboj/16.11.2985 PMC1169917

[8] Jamin Y, Tucker ER, Poon E, Popov S, Vaughan L, et al (2013) Evaluation of Clinically Translatable MR Imaging Biomarkers of Therapeutic Response in the Th-MYCN Transgenic Mouse Model of Neuroblastoma. Radiology 266: 130–140. 23169794 10.1148/radiol.12120128 PMC4298658

[9] Zhu S, Lee JS, Guo F, Shin J, Perez-Atayde AR, et al (2012) Activated ALK collaborates with MYCN in neuroblastoma pathogenesis. Cancer Cell 21: 362–373. 22439933 10.1016/j.ccr.2012.02.010 PMC3315700

[10] Heukamp LC, Thor T, Schramm A, De Preter K, Kumps C, et al (2012) Targeted expression of mutated ALK induces neuroblastoma in transgenic mice. Science translational medicine 4: 141ra191. 10.1126/scitranslmed.3003967 22764207

[11] Berry T, Luther W, Bhatnagar N, Jamin Y, Poon E, et al (2012) The ALK(F1174L) mutation potentiates the oncogenic activity of MYCN in neuroblastoma. Cancer Cell 22: 117–130. 22789543 10.1016/j.ccr.2012.06.001 PMC3417812

[12] Workman P, Aboagye EO, Balkwill F, Balmain A, Bruder G, et al (2010) Guidelines for the welfare and use of animals in cancer research. Br J Cancer 102: 1555–1577. 20502460 10.1038/sj.bjc.6605642 PMC2883160

[13] Walker-Samuel S, Orton M, McPhail LD, Boult JK, Box G, et al (2010) Bayesian estimation of changes in transverse relaxation rates. Magn Reson Med 64: 914–921. 20806382 10.1002/mrm.22478

[14] D'agostino RB (1986) Tests for normal distribution. In: R. B. D'agostino and M. A. Stepenes, editors. Goodness-of-fit techniques. New York, NY: Macel Decker. pp. 367–413.

[15] Goo HW (2010) Whole-body MRI of neuroblastoma. Eur J Radiol 75: 306–314. 19781884 10.1016/j.ejrad.2009.09.014

[16] Brisse HJ, McCarville MB, Granata C, Krug KB, Wootton-Gorges SL, et al (2011) Guidelines for imaging and staging of neuroblastic tumors: consensus report from the International Neuroblastoma Risk Group Project. Radiology 261: 243–257. 21586679 10.1148/radiol.11101352

[17] Robinson SP, Rijken PF, Howe FA, McSheehy PM, van der Sanden BP, et al (2003) Tumor vascular architecture and function evaluated by non-invasive susceptibility MRI methods and immunohistochemistry. J Magn Reson Imaging 17: 445–454. 12655584 10.1002/jmri.10274

[18] McDonald DM, Choyke PL (2003) Imaging of angiogenesis: from microscope to clinic. Nat Med 9: 713–725. 12778170 10.1038/nm0603-713

[19] Comstock JM, Willmore-Payne C, Holden JA, Coffin CM (2009) Composite pheochromocytoma: a clinicopathologic and molecular comparison with ordinary pheochromocytoma and neuroblastoma. American journal of clinical pathology 132: 69–73. 19864235 10.1309/AJCPN76VTIGWPOAG

[20] Robinson SP (2006) Blood oxygenation level dependent (BOLD) imaging of tumours. In: A. R. Padhani and P. L. Choyke, editors. New Techniques in Oncologic Imaging. Boca Raton: Taylor & Francis. pp. 257–272.

[21] Baluk P, Hashizume H, McDonald DM (2005) Cellular abnormalities of blood vessels as targets in cancer. Curr Opin Genet Dev 15: 102–111. 15661540 10.1016/j.gde.2004.12.005

[22] Christen T, Lemasson B, Pannetier N, Farion R, Remy C, et al (2012) Is T2* enough to assess oxygenation? Quantitative blood oxygen level-dependent analysis in brain tumor. Radiology 262: 495–502. 22156990 10.1148/radiol.11110518 PMC3267079

[23] Abramsson A, Berlin O, Papayan H, Paulin D, Shani M, et al (2002) Analysis of mural cell recruitment to tumor vessels. Circulation 105: 112–117. 11772885 10.1161/hc0102.101437

[24] Tatum JL, Kelloff GJ, Gillies RJ, Arbeit JM, Brown JM, et al (2006) Hypoxia: Importance in tumor biology, noninvasive measurement by imaging, and value of its measurement in the management of cancer therapy. Int J Radiat Biol 82: 699–757. 17118889 10.1080/09553000601002324

[25] McPhail LD, Robinson SP (2010) Intrinsic susceptibility MR imaging of chemically induced rat mammary tumors: relationship to histologic assessment of hypoxia and fibrosis. Radiology 254: 110–118. 20032145 10.1148/radiol.2541090395

[26] Hoskin PJ, Carnell DM, Taylor NJ, Smith RE, Stirling JJ, et al (2007) Hypoxia in prostate cancer: Correlation of BOLD-MRI with pimonidazole immunohistochemistry: initial observations. Int J Radiat Oncol Biol Phys 68: 1065–1071. 17637389 10.1016/j.ijrobp.2007.01.018

[27] Di Paolo D, Ambrogio C, Pastorino F, Brignole C, Martinengo C, et al (2011) Selective therapeutic targeting of the anaplastic lymphoma kinase with liposomal siRNA induces apoptosis and inhibits angiogenesis in neuroblastoma. Molecular therapy: the journal of the American Society of Gene Therapy 19: 2201–2212. 21829174 10.1038/mt.2011.142 PMC3242652

[28] Chantrain CF, Shimada H, Jodele S, Groshen S, Ye W, et al (2004) Stromal matrix metalloproteinase-9 regulates the vascular architecture in neuroblastoma by promoting pericyte recruitment. Cancer Res 64: 1675–1686. 14996727 10.1158/0008-5472.can-03-0160

[29] Chen Y, Takita J, Choi YL, Kato M, Ohira M, et al (2008) Oncogenic mutations of ALK kinase in neuroblastoma. Nature 455: 971–974. 18923524 10.1038/nature07399

[30] George RE, Sanda T, Hanna M, Frohling S, Luther W 2nd, et al (2008) Activating mutations in ALK provide a therapeutic target in neuroblastoma. Nature 455: 975–978. 18923525 10.1038/nature07397 PMC2587486

[31] Mosse YP, Laudenslager M, Longo L, Cole KA, Wood A, et al (2008) Identification of ALK as a major familial neuroblastoma predisposition gene. Nature 455: 930–935. 18724359 10.1038/nature07261 PMC2672043

[32] Janoueix-Lerosey I, Lequin D, Brugieres L, Ribeiro A, de Pontual L, et al (2008) Somatic and germline activating mutations of the ALK kinase receptor in neuroblastoma. Nature 455: 967–970. 18923523 10.1038/nature07398

[33] Fox E, Aplenc R, Bagatell R, Chuk MK, Dombi E, et al (2010) A phase 1 trial and pharmacokinetic study of cediranib, an orally bioavailable pan-vascular endothelial growth factor receptor inhibitor, in children and adolescents with refractory solid tumors. J Clin Oncol 28: 5174–5181. 21060028 10.1200/JCO.2010.30.9674 PMC3020690

[34] Carpenter EL, Haglund EA, Mace EM, Deng D, Martinez D, et al (2012) Antibody targeting of anaplastic lymphoma kinase induces cytotoxicity of human neuroblastoma. Oncogene 31: 4888. 10.1038/onc.2011.647 PMC3730824 22266870

[35] Carpenter EL, Mosse YP (2012) Targeting ALK in neuroblastoma—preclinical and clinical advancements. Nat Rev Clin Oncol 9: 391–399. 22585002 10.1038/nrclinonc.2012.72 PMC3683972

